# The Role of Dispositional Rule-Following and Metaphors About Psychological Flexibility on Operant Schedule Control

**DOI:** 10.3390/bs15121609

**Published:** 2025-11-22

**Authors:** Grace A. Lyons, Robert D. Zettle

**Affiliations:** Department of Psychology, Wichita State University, Wichita, KS 67260, USA; robert.zettle@wichita.edu

**Keywords:** tracking, pliance, metaphors, schedule control, psychological flexibility

## Abstract

Metaphors are used throughout acceptance and commitment therapy (ACT) to minimize the inflexibility of rule-governed, rather than contingency-shaped, behavior. Within the behavior analytic literature underlying ACT, responding on operant schedules has been used to parse out these differing sources of behavioral control. We thus used this preparation to more directly link the therapeutic use of metaphors to this literature. Participants were 105 undergraduates presented one of three passages—two metaphors and one nonmetaphor—with varying relevance for schedule control on an operant task where points could be both gained and lost. Schedule control was analyzed by visual analysis of cumulative point records over the course of the task. Two measures of dispositional rule-following—tracking and pliance—were also examined as moderators. No differences in schedule control were found between passage conditions alone. However, participants high in tracking who received the task-relevant metaphor were most likely to demonstrate schedule control reflective of psychological flexibility, while those low in both tracking and pliance who received the task-relevant metaphor were least likely to do so. Findings suggest dispositional tracking heightens the impact of therapeutic metaphors on psychological flexibility. Limitations and implications for further research on the behavior analysis of therapeutic metaphors are discussed.

## 1. Introduction

Acceptance and commitment therapy (ACT; Hayes et al., 2012b) has long emphasized using metaphors to decrease psychological inflexibility and promote psychological flexibility. Within ACT, a major contributing factor to psychological inflexibility is rigid adherence to rules about how to minimize unwanted experiences rather than accepting them for what they are (Hayes et al., 2012b). Conversely, psychological flexibility is more likely to occur when rules that become unworkable in producing lives worth living are abandoned (Hayes et al., 2012b).

Metaphors serve a central role in ACT because, as conceptualized by relational frame theory (RFT; Hayes et al., 2001), they provide a means of weakening the inflexibility attributable to literal language and many instances of rule-following. Stated differently, metaphors serve as experiential vehicles to promote flexible behavior that is more sensitive to its direct consequences rather than proposing more rules to rigidly follow in facilitating clinically relevant behavioral change. The superordinate take-home message to clients from metaphors within ACT to, in effect, “get out of your mind and into your life” (Hayes & Smith, 2005) is empirically supported by a meta-analysis of component studies that found the use of metaphors and other experiential exercises to be at least equally, if not more effective than related rationales alone in initiating self-reported change in key psychological flexibility and therapeutic processes (Levin et al., 2012).

A recognizable gap, however, remains in documenting and more fully understanding the effects of metaphors on observable behavior that exemplifies psychological flexibility. One such context for further investigating the impact of metaphors on psychological flexibility involves operant schedule control. While the behavior of nonhuman animals has been shown to be relatively sensitive to changes in and other features of programed schedules of reinforcement (e.g., Cole, 2001), the same has not always been true for humans (e.g., Delfabbro et al., 2023; Lowe et al., 1978), with human language cited as the most widely suspected contributing factor (Lowe & Horne, 1996).

The current study thus sought to analyze and understand the relative roles of metaphors and rule-following in altering human behavior in a psychologically flexible manner on operant schedules within a conceptual framework provided by contextual behavioral science (Hayes et al., 2012a). More specifically, it seems reasonable to posit that psychological flexibility/inflexibility is reflected by operant schedule control, or the degree to which responding is controlled by features of the schedules rather than instructions or rules about them, whether self-generated or researcher-provided (Galizio, 1979). Demonstrating schedule control would entail, for example, shifting from rapid to slower pressing of a button on a computerized task if and when doing so produces more points. Under such conditions, individuals may notice the unworkability of rapid responding and try alternative approaches, thereby transitioning from rigid rule following to actions that are more flexibly controlled by the direct consequences of their actions (Skinner, 1969).

While the literature on the clinical use of metaphors lacks the precision and rigor of experimentally analyzing variables that control behavior in the laboratory, much of human operant research neglects to examine the effects of analogue clinical interventions or dispositional characteristics on schedule performance, with only a few studies doing so to date (e.g., Chen & Reed, 2021; Reed, 2023). Therefore, this study aimed to experimentally investigate a contextual behavioral analytic account of the impact of metaphors and dispositional rule-following on schedule control as a proxy of psychological flexibility. To do so, this project evaluated the impact of three verbal presentations as the independent variable: (a) a task-relevant metaphor suggesting an accurate rule to follow in responding on a computerized task, (b) a task-irrelevant metaphor, and (c) task-irrelevant information. The major dependent variable was performance reflective of schedule control on a computerized operant task in which participants could gain or lose points on a single-alternative DRL schedule with a superimposed VR punishment schedule. This preparation was similar to that used by Bradshaw et al. (1979), such that rapid responding on a single manipulandum resulted in an accelerated loss of points via a variable ratio (VR) schedule, whereas slow responding on the same manipulandum resulted in the accumulation of points according to a differential reinforcement of low rate responding (DRL) schedule.

Much of the literature on rule-governed behavior and schedule control has utilized two different preparations. One preparation involves using multiple schedules to analyze differences in response rates between them (e.g., Chen & Reed, 2024; Reed, 2023; Wulfert et al., 1994). The other focuses on applying the generalized matching law (Baum, 1974; Méndez, 2024), where response rates on concurrent VI schedules tend to vary as a function of the rate of reinforcement provided by each schedule. “Schedule sensitivity” has been used in the former preparation to describe the occurrence of changes in responding that maximize reinforcement delivery, while sensitivity in the literature examining the generalized matching law refers to a mathematical parameter representing the behavioral responsiveness to a reinforcement ratio (Baum, 1974). Although both of the aforementioned preparations have been effective laboratory designs, they may lack generalizability to clinically relevant responding. In this project, we therefore opted to use a single-alternative DRL schedule with a superimposed VR punishment schedule using a single manipulandum insofar as it more closely parallels human responding to private events, and thus better elucidates psychological inflexibility. For example, if points earned in the task were to serve as a metaphor for desired thought-induced affective states, one could respond similarly on a VR schedule by “thinking more” in an attempt to elicit those experiences. However, consistent with ACT, such experiential control efforts paradoxically may only serve to push the targeted mood states further away (Swails et al., 2016). Alternatively, one could respond to pleasant private events in a more paced manner, an approach identified as experience prolonging (Swails et al., 2016). Although such behavioral shifts can be likened to schedule sensitivity as it is defined in multiple-schedule experiments, because the operant schedule in the current study differs significantly from its predecessors, we use the term schedule control to decrease confusion between concepts. Additionally, the current study dichotomizes the occurrence of schedule control to prioritize the occurrence of behavioral shifts toward reinforcement acquisition that represents flexibility rather than analyzing a continuous measure of sensitivity as is seen in the literature on the generalized matching law.

Although a main effect on schedule control for metaphor type was anticipated, individual differences were also expected. For example, Hayes et al. (1986) found that while many human subjects demonstrate at least some degree of schedule control (or what they describe as “schedule sensitivity”) when provided vague task instructions, an estimated 47%, who have been categorized as “nondifferential responders,” do not. It has been suggested that both those who demonstrate schedule control and nondifferential responders are likely, as verbal organisms, to be engaging in rule-governed behavior, although of different functional classes (Hayes et al., 1986; Zettle & Hayes, 1982). One such class, pliance, refers to rule-following under the dual control of natural consequences of engaging in the actions specified by the rule as well as arbitrary, socially mediated consequences for complying with the rule (Zettle & Hayes, 1982). Tracking, as a second class of rule-following, refers to behavior only under the control of the natural consequences for following the rule or instruction in question (Zettle & Hayes, 1982).

To better predict individual differences in schedule control, Wulfert et al. (1994) examined dispositional cognitive rigidity, which can be seen as a proxy of pliance, as a moderating variable. Those who self-reported more cognitive rigidity generally demonstrated lower schedule control. Over the last few years, self-report measures of dispositional tendencies to engage in pliance versus tracking have been developed (Inoue & Okajima, 2025; Ruiz et al., 2019, 2020) that may provide greater promise as moderators in accounting for variability in schedule control. This study additionally sought to more precisely examine the degree to which individual differences in dispositional rule-following, specifically generalized pliance (Ruiz et al., 2019) and generalized tracking (Ruiz et al., 2020), might moderate schedule control. It was expected that those reporting higher levels of generalized tracking, rather than pliance, would demonstrate greater schedule control considering it represents more flexible rule-governance, especially when also presented with the task-relevant metaphor. The interaction of tracking and pliance on schedule control was also explored but in a more exploratory nature.

This study has the potential to make several contributions to the literature regarding the use of psychotherapeutic metaphors, particularly within ACT. First, confirmatory findings concerning the independent variable would provide empirical support that metaphors have a desirable effect on overt behavior in addition to thoughts and emotions, thereby providing further support for their continued use as therapeutic tools. Confirmatory findings about the moderating effect of dispositional rule-following would ostensibly have both conceptual and clinical implications for the use of psychotherapeutic metaphors. Conceptually, confirmatory findings would further our understanding of the interrelationship among metaphors, rule-governed behavior, and psychological flexibility. Pragmatically, findings suggestive of moderating roles for dispositional pliance and tracking would provide further support for the development and utilization of therapeutic metaphors that address these types of rule-governed behavior.

## 2. Materials and Methods

### 2.1. Participants

This study was approved by an institutional review board and followed the ethical principles of the American Psychological Association (2017). Participants consisted of 105 undergraduate students from a Midwestern university who met eligibility requirements of being fluent in English and over the age of 18. The sample had a mean age of 20.98 years (SD = 6.26) and were predominantly White (69.5%), non-Latine (81%), and female (68.6%). No differences in any demographic or moderating variables were found between conditions.

### 2.2. Moderating Variables

#### 2.2.1. Generalized Pliance Questionnaire—9

The Generalized Pliance Questionnaire—9 (GPQ-9; Ruiz et al., 2019) is a shortened 9-item version of the Generalized Pliance Questionnaire designed to assess a dispositional tendency to engage in rule-following where the predominant reinforcer is social approval (e.g., “To feel good about myself, I need other people’s approval;” Ruiz et al., 2019). Items are rated on a 7-point Likert scale from 1 (*never true*) to 7 (*always true*), resulting in a total score of 9–63, in which higher scores indicate elevated levels of generalized pliance. The GPQ-9 has demonstrated good internal consistency (α = 0.91–0.95; Ruiz et al., 2019) and has been positively correlated with measures of experiential avoidance, cognitive fusion, and distressing emotional symptoms (Ruiz et al., 2019). Cronbach’s α in the current study was 0.90, indicating good internal consistency.

#### 2.2.2. Generalized Tracking Questionnaire

The Generalized Tracking Questionnaire (GTQ; Ruiz et al., 2020) is an 11 item self-report measure designed to assess the dispositional tendency to engage in rule-following under the control of its direct and natural consequences (e.g., “I make decisions based on my experience and not what others say;” Ruiz et al., 2020). Items are rated on a 7-point Likert scale from 1 (*never true)* to 7 (*always true*), resulting in a total score of 11–77, in which higher scores indicate increased levels of generalized tracking. The GTQ has demonstrated good internal consistency (α = 0.85–0.87; Ruiz et al., 2020; Stapleton et al., 2022) and has been shown to be sensitive to clinical status (Ruiz et al., 2020). As noted, it negatively correlates with generalized pliance, experiential avoidance, cognitive fusion, repetitive thinking, and emotional symptoms (Ruiz et al., 2020). Conversely, it positively correlates with progress in values, satisfaction with life, and general self-efficacy (Ruiz et al., 2020). Cronbach’s α in the current study was 0.88, reflecting good internal consistency.

### 2.3. Operant Task

Participants were told the purpose of the project was to collect normative data on a newly developed computerized task. They were seated in a research laboratory in front of a desktop computer on which the task was presented. Specialized software was programmed using Visual Studio 2022 version 17.7.3 to present stimuli and register clicks on a computer mouse. The task was structured using a single-alternative DRL schedule and a superimposed VR punishment schedule with a single manipulandum by which participants could gain and lose points. Participants received the following instructions presented visually on the screen at the beginning of the task, with no further printed or verbal instructions provided during the task:

Your goal in this task is to end with as many points as possible. You will start with 200 points. Each point represents a chance to earn one of three $15 gift cards. Points are determined by clicking the “Points” button with the mouse on the next page. The task will last for 20 min, after which you will see the point counter turn dark red and read “Final Points.” Alert the experimenter when the task ends.

Click the “Start” button to begin.

Following the presentation of the instructions, the experimenter left the room to minimize pliance. Clicking the “Start” button produced a new screen displaying a box reading “Current Points: 200” in its center and a separate box labeled “Points” directly below it. The “Points” button was programmed to flash blue when clicked to signal that a response was registered. Other visual stimuli (e.g., the “Current Points” box) did not register responses or change color when clicked to discourage extraneous clicking outside of the “Points” button.

The point count decreased by 1 point on a VR 10 (7–13) schedule and increased by 5 points on a DRL 15 s schedule based on clicking the “Points” button. This point differential was consistent with similar studies utilizing comparable schedules so points gained always outweighed points lost to encourage continued responding (e.g., Chen & Reed, 2021; Schlund et al., 2017). Any mouse click on the “Points” button less than 15 s apart resulted in the 15 s timer resetting. When a click resulted in a change in points, the point counter turned red for 0.5 s to denote a loss of a point or green for 0.5 s to denote the gaining of points. If a click simultaneously activated both the point loss and point gain schedules, the point counter increased by 4 points and turned green to denote a net increase. The program ran uninterrupted for 20 min, after which the “Current Points” box turned red and read “Final Points: ____.”

#### Task-Related Measures

Data collected during the task included the current point count at each second and the second at which a mouse click occurred on the “Points” button. Any responses outside of the “Points” button were not recorded. These data were automatically downloaded into a .csv file by the program upon task conclusion.

**Figure 1:**
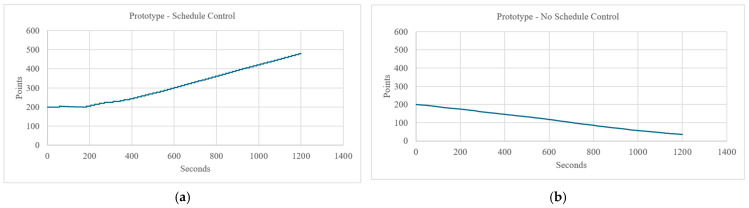
Prototypical graphs of points over time given to raters to make judgements of schedule control. Panel (**a**) represents a cumulative record demonstrating schedule control. Panel (**b**) represents a cumulative record failing to demonstrate schedule control.

Cumulative records were created displaying a participant’s point count at each second of the task. Individual cumulative records were then judged for demonstrated schedule control by two independent raters blind to their assigned intervention. The judges were provided exemplar cumulative records (See Figure 1) and written instructions for determining schedule control to aid in their visual analyses. Schedule control was demonstrated by a change from point loss to consistent point gains over the duration of the task. Lack of schedule control was exemplified by a failure to steadily earn points by decreasing response rates during the task. The raters had a 97% agreement rate (*n* = 102). The three disagreements between judges were settled by a third, independent, blind evaluator.

### 2.4. Passage Conditions^1^

Participants were randomly assigned to one of three conditions—one experimental and two control groups—and informed their participation was to obtain normative data on a newly developed reading comprehension task. The experimental group (*n* = 35) received a task-relevant metaphor, while the control groups received either a task-irrelevant metaphor (*n* = 37) or task-irrelevant information (*n* = 33). All passages were presented in writing on a computer screen while being spoken through headphones. Passages ranged from 391 to 408 words and from 2 min to 2 min 14 s when recorded. Both metaphors, based on a recommendation by Hayes et al. (2012b), were written in present tense from the participant’s point of view.

#### 2.4.1. Task-Relevant Metaphor

Participants in this condition were presented an extended metaphor in which they were asked to imagine being gifted a plant at a housewarming party. The metaphor was designed to illustrate psychological inflexibility and flexibility, as well as pliance and tracking, by demonstrating that overwatering of a plant for it to grow faster not only prevents its growth but actively makes the situation worse. The take-home message from this metaphor was designed to be directly applicable to the operant task previously described in which responding at a slow, steady pace optimally earns points.

#### 2.4.2. Task-Irrelevant Metaphor

Participants in this condition were presented with an extended metaphor in which they were asked to imagine taking a middle school math class and their parent steps in to solve a difficult math problem for them. The metaphor was designed to illustrate the sometimes necessary and useful stance of persistence in the face of struggling circumstances. This take-home message, however, was expected to be irrelevant to performance on the operant task.

#### 2.4.3. Task-Irrelevant Information

Participants in this group were presented information about uncontrollable worrying and the role of positive and negative beliefs about it (Robinson et al., 2023) to serve as a control condition in which participants neither received a metaphor, nor was the take-home message from the passage related to the operant task.

### 2.5. Procedure

Participants used an online platform to sign up for a 45 min timeslot to complete the study in-person with a trained researcher. The time allotted to complete the study was deemed sufficient during pilot trails. To better understand the natural carry-over effects of metaphor exposure, participants were told their participation was in service of gathering normative data on four recently developed measures/tasks for use in subsequent research: (a) a battery of personality inventories, (b) an executive task of cognitive functioning, (c) a reading comprehension task, and (d) a computerized task. The presentation order of each study component did not vary between participants.

After providing informed consent and placing any cell phones, watches, and similar electronic devices into a designated container, participants completed a demographic questionnaire. Participants then completed the first component of the overall study, during which the GTQ, the GPQ-9, and the NEO Personality Inventory-3 (NEO-PI-3; McCrae et al., 2005) as a mask were administered via Qualtrics. Participants then completed Part B of the Trail Making Test (Army Individual Test Battery, 1944) as a second mask for the overall purpose of the study.

The third part of the study involved presentation of the randomly assigned passages in writing and via audio-recording through headphones. Immediately afterwards, participants responded to several questions designed to assess their understanding of the passage’s take-home message and attitudes towards it. More specifically, participants were asked to choose from a set of four options which take-home message best fit the passage they were presented. The following options were provided: (a) “sometimes we are better off doing less than doing more” (task-relevant metaphor), (b) “overcoming struggles is sometimes necessary to make us stronger” (task-irrelevant metaphor), (c) “worrying itself is a bigger problem than what you are worried about” (task-irrelevant information), and (d) “we can lose sight of what’s happening now by focusing on the past” (none of the passages). These response options were verified by study collaborators as accurate representations of the passages and as each applying to only one passage. Participants then completed the computerized operant task as the last component of the study, after which they answered questions regarding the task instructions and whether they followed them to serve as a manipulation check prior. Finally, participants were given a debriefing form that continued to describe the normative data-gathering purpose of the study in addition to providing contact information for the researchers.

## 3. Results

### 3.1. Attention and Manipulation Checks

There were significant differences between passages in participants’ understanding of their take-home messages. Specifically, when asked to indicate the correct take-home message from a set of options, significantly fewer participants who received the task-relevant metaphor (*n* = 23 or 65.7%) selected the correct one compared to those receiving the task-irrelevant metaphor (*n* = 33 or 89.2%) or task-irrelevant information (*n* = 32 or 97%), *ꭓ*^2^ (2, *N* = 105) = 13.45, *p* = 0.001. When data from the other two conditions were included, those who did not correctly select their take-home message reported significantly higher levels of dispositional pliance based on GPQ-9 scores (*M* = 37.18 vs. 31.40), *t*(103) = 2.21, *p* = 0.03, *d* = 0.59.

When asked about the instructed goal of the operant task, participants could select from options that were either verbatim correct (i.e., To end with as many points as possible), functionally correct (i.e., To gain as many points as possible), or incorrect (i.e., To lose as many points as possible). Most participants selected one of the correct options (*n* = 97 or 92.4%), with the proportion of those doing so not differing across the three groups, *ꭓ*^2^ (2, *N* = 105) = 0.40, *p* = 0.82. They did not differ in GPQ-9 scores, *t*(103) = 0.90, *p* = 0.37 from their counterparts who failed to do so but did report higher GTQ scores with a large effect size, *t*(103) = 2.28, *p* = 0.03, *d* = 0.84.

### 3.2. Visual Analysis of Cumulative Records

When dichotomously rated, 73.3% (*n* = 77) of the cumulative records of points earned during the task were judged as demonstrating schedule control. There were no significant differences between all three passages in the proportion of participants evidencing schedule control, *ꭓ*^2^ (2, *N* = 105) = 0.34, *p* = 0.84.

#### Microanalyses of Cumulative Records

Schedule control was further analyzed based on two microanalyses of cumulative records. The first of these was based on participants’ understanding of the passage take-home message and operant task instructions. There was no significant difference in the proportions who demonstrated schedule control between those who did and did not select the respectively correct passage take-home message regardless of group membership, *ꭓ*^2^ (1, *N* = 105) = 0.84, *p* = 0.36, or within any passage condition. Similarly, no difference in the proportions of participants displaying schedule control was found when analyzed by which of the four take-home message options they selected *ꭓ*^2^ (2, *N* = 105) = 2.02 *p* = 0.36. Participants were, however, more likely to demonstrate sensitivity if they selected either the verbatim or functionally correct task instructions, *ꭓ*^2^ (1, *N* = 105) = 23.81, *p* < 0.001, with no significant difference in likelihood of sensitivity when specifically comparing the verbatim and functionally correct options, *ꭓ*^2^ (1, *n* = 97) = 0.01, *p* = 0.93.

### 3.3. Dispositional Rule-Following

We considered two ways of analyzing the effects of passage condition and dispositional rule-following on schedule control. One method was to conduct a binary logistic regression, which would maintain the continuous nature of the GPQ-9 and GTQ and also include passage condition as variables in predicting schedule control as a dichotomous outcome. The primary disadvantage of this option, however, is that the our sample did not meet the events per variable criterion (Peduzzi et al., 1996) for conducting logistic regression models. That is, the smaller outcome group of 28 participants (27%) who did not demonstrate schedule control fell short of having at least 10 participants for each of the seven predictors in the model, thereby significantly increasing the risk of overfitting.

Consequently, we instead opted to dichotomize scores on the GPQ-9 and GTQ into high and low subgroups via median splits. This, too, presents disadvantages, such as decreased understanding of individual differences, potential changes to effect size and statistical power, and the possibility of overlooking nonlinear relationships (MacCallum et al., 2002). Despite these limitations, our chosen analytic option is supported by both historical (e.g., Wulfert et al., 1994) and more recent (e.g., Chen & Reed, 2021; Reed, 2019) practices of dichotomizing nonschedule-related predictor variables, such as dispositional measures, in studies of human operant performance and schedule control.

Consistent with other reports (i.e., Ruiz et al., 2020), scores from the GPQ-9 and the GTQ were modestly correlated, *r* = −0.30, *p* = 0.002. Importantly, the association between the GPQ-9 and GTQ was not so strong as to preclude the creation of subgroups for subsequent moderation analyses by conducting median splits on both measures. The 11 participants who scored at the GPQ median (33 of 63) were allocated to the high subgroup, yielding two roughly equivalent subgroups (high *n* = 54, low *n* = 51) that significantly differed from each other in their levels of dispositional pliance, *t*(103) = 13.02, *p* < 0.001, *d* = 2.54. Adding two participants scoring at the median (55 of 77) on the GTQ to the low subgroup created roughly equivalent subgroups (high *n* = 52, low *n* = 53) that significantly differed from each other in their levels of dispositional tracking, *t*(103) = 14.36, *p* < 0.001, *d* = 2.80.

The following four subgroups were created by the two median splits: (a) high pliance/high tracking (*n* = 21), (b) high pliance/low tracking (*n* = 33), (c) low pliance/high tracking (*n* = 31), and (d) low pliance/low tracking (*n* = 20). The distribution of participants significantly differed across the four subgroups, *ꭓ*^2^ (1, *N* = 105) = 5.03, *p* = 0.03, as they were more likely to fall into one of the two high/low subgroups on both measures. This is not surprising given the moderately inverse relationship between the GPQ-9 and GTQ.

#### 3.3.1. Effects of Tracking

Results displayed in Table 1 revealed those in the high tracking group (43 of 52 = 82.7%) were significantly more likely to demonstrate schedule control than those in the low tracking group (34 of 53 = 64.1%), *ꭓ*^2^ (1, *N* = 105) = 4.61, *p* = 0.03. The moderating effect of dispositional tracking, however, became interactional when separately analyzed by passage conditions. There was a moderating effect of tracking only for the task-relevant metaphor group, *ꭓ*^2^ (1, *n* = 35) = 4.99, *p* = 0.03, in which those with higher GTQ scores were more likely to display schedule control (14 of 15 = 93.3%) compared to their lower scoring counterparts (12 of 20 = 60%). The effect did not hold for the task-irrelevant metaphor, *ꭓ*^2^ (1, *n* = 37) = 1.11, *p* = 0.29, or task-irrelevant information conditions, *ꭓ*^2^ (1, *n* = 33) = 0.17, *p* = 0.68, even though participants in both groups with higher levels of dispositional tracking were relatively more likely to show schedule control (18 of 22 = 81.8% and 11 of 15 = 73.3%, respectively).

#### 3.3.2. Effects of Pliance

No difference in likelihood of demonstrating schedule control was found based on high (43 of 54 = 79.6%) or low levels of pliance (34 of 51 = 66.7%), *ꭓ*^2^ (1, *N* = 105) = 2.25, *p* = 0.13. There was also no interaction between level of dispositional pliance and passage conditions.

#### 3.3.3. Additional Interactive Effects

When collapsed across passage conditions, there was a significant two-way interaction between pliance and tracking on schedule control as assessed by a visual analysis of cumulative records, *ꭓ*^2^ (3, *N* = 105) = 10.80, *p* = 0.01 (See Table 2). More specifically, those within the low pliance/low tracking subgroup were less likely to demonstrate schedule control (9 of 20 = 45%) than those in the other three subgroups in which at least 75% of participants displayed sensitivity.

In assessing for a possible three-way interaction between the four pliance/tracking subgroups and passage conditions, a significant moderating effect was found for the task-relevant metaphor condition alone, *ꭓ*^2^ (3, *n* = 35) = 8.75, *p* = 0.03, in which the proportion of participants within its low pliance/low tracking subgroup who displayed schedule control was significantly lower (3 of 8 = 37.5%) than that of the other three subgroups combined (23 of 27 = 85.2%). This finding parallels that reported in the preceding paragraph and accounts for the overall interactive and moderating effect of dispositional rule-following when collapsing across passage conditions.

## 4. Discussion

The current study sought to examine the role of metaphors and dispositional rule-following on overt behavior during an operant task in which schedule control served as a proxy for psychological flexibility. Additionally, we sought to add empirical support of the use of therapeutic metaphors using an analogue preparation. Consistent with the tradition of behavior analytic research, cumulative records of points earned during the operant task were analyzed. Our primary hypothesis was that participants presented a metaphor with a take-home message about optimally responding at a slow, steady pace would be more likely to demonstrate schedule control than those who received either of the two control passages. This hypothesis was largely unsupported, as no differences were found between any of the passages.

In examining the degree to which generalized tracking and pliance moderate the impact of passage conditions on schedule control in concordance with the findings of Wulfert et al. (1994) about dispositional cognitive rigidity, findings supported a moderating role of tracking on schedule sensitivity, particularly for the task-relevant metaphor. This finding, which was anticipated yet more powerful than expected, suggests a dispositional tendency toward tracking not only adds to responding flexibly and effectively to novel, ambiguous situations, but these effects can be facilitated by the use of metaphors from which potentially relevant rules for responding might be derived. The notion that those with higher levels of tracking were better able to follow instructions is further supported by the findings that higher GTQ scores were found among participants who selected at least the functionally correct task instructions. In short, those high in dispositional tracking appeared to be more attentive to information about the task and more able to flexibly use this information to guide their behavior. This finding and related interpretation highlights the importance of assessing self-reported generalized tracking at the onset of clinical treatment. Newly developed measures, such as the Generalized Pliance and Tracking 2-way Scale (GPT-2s; Inoue & Okajima, 2025) may help further elucidate the role of dispositional rule-following, as it assesses both pliance and tracking across eight everyday situations. Future research may also seek to better understand how tracking abilities can be improved in order to make therapeutic metaphors more effective in producing behavioral change. Additionally, the findings in the current study would be strengthened by future studies obtaining sample sizes with a sufficient number of participants who fail to demonstrate schedule control, such that binary logistic regression can be employed to more precisely predict schedule control.

While a main effect of pliance was not found, an interaction between the two types of rule-following was found in which those low in both pliance and tracking were less likely to demonstrate schedule control. Again, this relationship was particularly strong for those receiving the task-relevant metaphor. This seems to represent further evidence for the interpretations regarding the main effect of tracking. That is, lower generalized tracking appears to represent a disinclination to derive and flexibly follow instructions based upon the natural consequences of doing so. Contrary to expectations was the interaction with low levels of generalized pliance. Low scores on the GPQ-9, when combined with low tracking, may most readily be construed as indicating a tendency to simply not follow what are seen as directives. Another possibility, however, is that low levels of generalized pliance might also reflect reactive disobedience known as counterpliance (Hayes et al., 1989).

Like pliance, counterpliance is socially mediated rule-following, but in which the resulting behavior is the *opposite* of that specified by the rule (Hayes et al., 1989). Stated differently, an individual may behave in ways they are specifically told not to or defiantly opposite to what they are instructed. To illustrate, when given the rule to wear a mask in public during the COVID-19 pandemic, high pliance would entail doing so in many, if not most, situations because it is at least partially socially reinforced. Low pliance would likely be reflected by at least some mask wearing, but to a significantly lesser degree than that exhibited by their fellow citizens high in dispositional pliance. By contrast, counterpliance would be exemplified by consistent and adamant refusal to wear a mask in recommended situations (Stapleton, 2020). In the context of the current study, those low in tracking may have been less likely to derive potentially relevant rules from the task-relevant metaphor to guide their behavior during the task. If those who also had low pliance scores could be supposed to have higher levels of counterpliance, then any rules potentially derived from the metaphor may have supported behavior in direct opposition to them, leading to a further decreased likelihood of schedule control.

Unfortunately, no self-report measures of counterpliance similar to the GPQ-9 have been developed to empirically evaluate its relationship with pliance. However, means of assessing a similar concept in social psychological research, psychological reactance (i.e., a motivational state brought about by a perceived threat to behavioral freedom; Brehm, 1966), have been developed. While the current study did not employ measures that might have captured counterpliance or similar constructs, future studies may wish to include such instruments.

### Limitations and Future Directions

While the current study shed light on the moderating effect of dispositional rule-following on the impact of therapeutic metaphors in producing operant schedule control, several limitations remain. Among limitations to internal validity are potential issues related to the lack of comparison between the effects of derived and direct instructions on schedule control. While those high in tracking seemed to be more likely to derive and follow instructions, it remains unclear how these participants would have responded to direct instructions, which tend to be followed more quickly and more rigidly than derived instructions (Harte et al., 2017). Future studies should thus include a direct instruction comparison group to better understand the utility of dispositional tracking.

Several other limitations pertain to external validity. Importantly, although the operant task in this project sought to more closely resemble clinically relevant situations, the overall methodology had notable differences compared to therapeutic contexts in which metaphors might be used. Notably, a convenience sample of college students was used, which may impact rule-following in several ways. For instance, it remains unclear the degree to which distress impacts one’s ability to derive rules, let alone apply them in potentially counterintuitive ways (i.e., “Less is more”). At least some evidence suggests that psychiatric symptoms like depression impact a person’s performance on operant schedule (Chen & Reed, 2021). It is advisable that future studies utilize clinical samples or minimally assess distress and/or other characteristics (e.g., self-efficacy) that may impact rule-following.

Another limitation is related to the generalizability of the operant task to clinical situations. The task in this project provided clear, visual feedback about the effects of responding (i.e., the point counter turned green and the ongoing total increased or turned red and decreased). The same may not necessarily be true for all clinical circumstances, particularly when reinforcement is less external and more arbitrary (e.g., a decrease in distress or increase in satisfaction). Such natural consequences may require individuals to be more attuned to emotional states and changes in them. If the person is not sensitive to these experiences, they may encounter difficulty engaging in tracking because they have not fully contacted the consequences of their responses. Future studies should therefore consider employing tasks that measure psychological consequences or ask participants to detail their evaluative reactions to them.

In conclusion, the limitations to the generalizability of findings from this project suggest future research would be enhanced by more closely mimicking clinical situations. This could be achieved by utilizing clinical or subclinical samples and exploring other predictors of schedule control, like self-reported counterpliance or self-efficacy.

## 5. Conclusions

Overall, the success of therapeutic metaphors may hinge on dispositional rule-following, particularly generalized tracking. Higher levels of generalized tracking, as expected, were related to a greater likelihood of demonstrating schedule control, especially for those presented the task-relevant metaphor. This suggests the use of metaphorical language may be broadly useful for clients with high levels of dispositional tracking, which itself may serve as a proxy for psychological flexibility. Clinicians may then capitalize on this dispositional tendency by providing metaphors from which relevant rules might be derived. The superordinate takeaway from this study, therefore, seems to be that baseline assessment of dispositional rule-following would be beneficial in understanding for whom therapeutic metaphors may be productive, unproductive, and even counterproductive.

## Figures and Tables

**Table 1:** Participants Displaying Schedule Control by Passage Conditions and Dispositional Rule-Following.

Condition	Task–Relevant Metaphor (*n* = 35)	Task–Irrelevant Metaphor (*n* = 37)	Task–Irrelevant Information (*n* = 33)	Total (*N* = 105)
Tracking				
Low	12/20 (60.0%)	10/15 (66.7%)	12/18 (66.7%)	34/53 (64.1%)
High	14/15 (93.3%)	18/22 (81.8%)	11/15 (73.3%)	43/52 (82.7%)
Pliance				
Low	11/17 (64.7%)	13/18 (72.2%)	10/16 (62.5%)	34/51 (66.7%)
High	15/18 (83.3%)	15/19 (78.9%)	13/17 (76.5%)	43/54 (79.6%)

**Table 2:** Percentage of Participants Displaying Schedule Control by Passage Conditions and Combinations of Dispositional Rule-Following.

Condition	Task-Relevant Metaphor (*n* = 35)		Task-Irrelevant Metaphor (*n* = 37)		Task-Irrelevant Information (*n* = 33)		Total (*N* = 105)	
Tracking	Low	High	Low	High	Low	High	Low	High
Pliance								
Low	3/8	8/9	3/6	10/12	3/6	7/10	9/20	25/31
	(37.5%)	(88.9%)	(50.0%)	(83.3%)	(50.0%)	(70.0%)	(45.0%)	(80.6%)
High	9/12	6/6	7/9	8/10	9/12	4/5	25/33	18/21
	(75.0%)	(100%)	(77.8%)	(80.0%)	(75.0%)	(80.0%)	(75.8%)	(85.7%)

## Data Availability

The data are available upon request from the corresponding author.

## Note

1 Copies of the passages can be accessed by contacting the first author.
